# Benefits of Mobile Apps in Pain Management: Systematic Review

**DOI:** 10.2196/11231

**Published:** 2018-10-22

**Authors:** Simon E Thurnheer, Isaac Gravestock, Giuseppe Pichierri, Johann Steurer, Jakob M Burgstaller

**Affiliations:** 1 Horten Centre for Patient Oriented Research and Knowledge Transfer University of Zurich Zurich Switzerland

**Keywords:** mobile application, pain, pain management, smartphone, cell phone, telemedicine, review

## Abstract

**Background:**

Pain is a common condition with a significant physical, psychosocial, and economic impact. Due to enormous progress in mobile device technology as well as the increase in smartphone ownership in the general population, mobile apps can be used to monitor patients with pain and support them in pain management.

**Objective:**

The aim of this review was to assess the efficacy of smartphone or computer tablet apps in the management of patients with pain.

**Methods:**

In December 2017, a literature search was performed in the following databases: MEDLINE, EMBASE, CINAHL, Cochrane, and PsycINFO. In addition, a bibliography search was conducted. We included studies with at least 20 participants per arm that evaluated the effects of apps on smartphones or computer tablets on improvement in pain.

**Results:**

A total of 15 studies with 1962 patients met the inclusion criteria. Of these, 4 studies examined the effect of mobile apps on pain management in an in-clinic setting and 11 in an out-clinic setting. The majority of the original studies reported beneficial effects of the use of a pain app. Severity of pain decreased in most studies where patients were using an app compared with patients not using an app. Other outcomes, such as worst pain or quality of life showed improvements in patients using an app. Due to heterogeneity between the original studies—patient characteristics, app content, and study setting—a synthesis of the results by statistical methods was not performed.

**Conclusions:**

Apps for pain management may be beneficial for patients, particularly in an out-clinic setting. Studies have shown that pain apps are workable and well liked by patients and health care professionals. There is no doubt that in the near future, mobile technologies will develop further. Medicine could profit from this development as indicated by our results, but there is a need for more scientific inputs. It is desirable to know which elements of apps or additional devices and tools may improve usability and help patients in pain management.

## Introduction

Mobile device technology has improved enormously in the past years. With higher screen resolution or better processor performance, not only has the hardware improved but also many software programs known as mobile apps have been developed, thereby setting up a technological revolution. Use of internet on smartphones and the proportion of total internet use have increased within the past few years [1-3] and are part of the daily life worldwide. The number of health-related mobile apps increased rapidly within the past 10 years; in 2017, about 40% of the more than 300,000 apps available on the market were related to health issues, including monitoring and management of illnesses [4]. Chronic illnesses seem to be a particular target for the use of these apps. The result of a review showed that the use of apps helps to improve asthma control and even lung function. Patients’ symptoms and medication usage are recorded, and recommendations to adapt treatment, for example, to increase the dose of inhaled drugs, are provided [5]. Patients with type 1 diabetes appreciate an app as a supplement for disease management (eg, communication with health care professionals) [6]. The use of a smartphone app shortens times for medical personnel to review glucose-level diaries from patients [7] and has the potential for improving glucose control in type 1 and 2 diabetes [7,8].

Pain, acute and chronic, is a substantial burden on individuals, health care systems, and employers [9]. Patients with acute pain need to be treated carefully to prevent abuse of pain medication, in particular the abuse of opioids, and to prevent the development of chronic pain. Apps assist to monitor patients with acute or chronic pain and can inform and support them in the management of pain, for example, changes in the dosage of analgesics, early detection of adverse effects of analgesics, or providing coping strategies to manage pain. Published (systematic) reviews appraised the usability of smartphone or computer tablet app use in patients with pain. The reviews show that many of the commercially available apps lack usability and have other limitations, such as absence of rigorous scientific evaluation of the provided content and the recommendations given to patients [10-17]. None of the published reviews assessed the efficacy of apps in the management of patients with acute or chronic pain. The objective of this review is to assess the efficacy of smartphone or computer tablet apps in the management of patients with pain.

## Methods

### Data Reporting

This systematic review is based on the preferred reporting items for systematic reviews and meta-analyses (PRISMA) [18].

### Literature Search

At the end of December 2017, a systematic literature search was commissioned to the Careum Bibliothek of the University of Zurich. The following electronic databases were searched by an experienced librarian: MEDLINE, EMBASE, CINAHL, Cochrane, and PsycINFO. We used, among others, the following search terms as medical subject headings and other subject headings: “Pain+,” “Pain Management,” “Pain Measurement,” “Cellular Phone+,” “Mobile Devices,” “Mobile Applications,” “Telehealth,” and “Telemedicine+.” Detailed description of one search strategy is provided in Multimedia Appendix 1. Only articles written in English or German were considered. No restriction regarding publication date was applied.

### Eligibility Criteria

Studies included in this review evaluated the effects of mobile apps (which we refer to as apps and are defined as a type of application software designed to run on a mobile device [19]) on smartphones or computer tablets on improvement in pain. Studies with at least 20 participants per arm were included and could have an in-clinic or an out-clinic setting. In-clinic setting describes the situation when the intervention with an app was made in a hospital, clinic, or another institution only, regardless of whether patients were in ambulatory or stationary treatment (being cared for at least 24 hours in a hospital). Out-clinic describes the setting when the intervention with an app was performed by the patients themselves in an ambulatory treatment, meaning patients could use the app wherever they were.

Studies were excluded if devices other than a smartphone or computer tablet (eg, smartwatches, palmtops, handheld computers, or similar devices) or if an app not defined as above was used for data collection (eg, internet website and short message service [SMS]). Furthermore, exclusion criteria were the use of apps designed for diagnosis of a medical condition or not explicitly designed for pain-level recording, management, or treatment (eg, usual music players, video conference programs, and video games). These exclusion criteria did not apply if there was another app that was designed to take part in the study, for example, pain diary. In addition, studies were excluded if they included patients with a cognitive handicap, did not provide sufficient baseline data, described the development process of an app without measuring the effects of app use on pain, were conducted in the field of veterinary health, or were not available for purchase.

### Study Selection and Data Extraction

All references were initially screened by title and abstract by 2 reviewers (SET and JMB) for relevance. Finally, full-text analysis for eligibility was performed by SET and JMB independently. Disagreements were discussed and resolved by consensus or third-party arbitration (JS).

### Outcomes

The primary outcome of interest of this systematic review was improvement of pain. Other outcomes of interest assessed in the studies, such as worst pain (the worst pain intensity during a certain defined observational period) or improvement in mobility were also included in analysis.

### Quality of Studies

The checklist of the Scottish Intercollegiate Guidelines Network (SIGN) [20] for randomized controlled trials (RCTs) was used to review the quality of included RCTs. Overall assessment of each study focused on bias minimization was rated in categories; RCT categories are high quality (++), acceptable (+), low quality (−), and reject (0). High ratings mean the majority of criteria are met and further research is unlikely to change results; acceptable quality is provided by studies in which most criteria are met with few flaws, but conclusions may change after future studies. Low quality is given if either most criteria are not met or significant flaws in key aspects of the study design are present, and therefore, further studies may change conclusions. If a study did not meet the SIGN quality criteria and was considered to be in the category unacceptable, it was rejected.

For before-after studies (often called pre-post studies where variables are measured before and after an intervention and all participants are assigned to 1 intervention group), no checklist is required [20].

### Statistics

The primary objective of this study was to assess the mean pain difference between intervention and control groups or before and after intervention. If data for several time points were available, the last one was considered to be the most relevant to our analysis. Heterogeneity of studies did not allow meta-analysis; so, only descriptive and comparative analysis was used to summarize findings across all studies.

A secondary objective was to summarize other outcomes as well as applicability and feasibility of the apps in a qualitative way. Most of the additional outcomes reviewed were too heterogeneous to compare statistically. Qualitative descriptive exploration was performed, and the most important results were summarized in a table.

For the primary comparative analysis, studies were included if sufficient data were available and measurement scales were comparable. Only study results with very similar pain rating scales were included (eg, Visual Analog Scale, VAS, on a scale of 0-10; numeric rating scale, NRS, 0-10; and VAS on a scale of 0-100), and where necessary, different pain score scales were rescaled to a 0- to 10-point scale. We graphically show the mean pain scores over time separately for each study arm where available. The effects of treatment in the multiple-arm studies with sufficient data for comparison are shown in a forest plot. Studies that did not have an observational period—all of which have an in-clinic study setting—are shown in a separate graph. If data were missing and could not be calculated from the other available data, corresponding authors were contacted twice via email. If authors did not respond, data were considered missing.

## Results

### Study Selection

**Figure 1:**
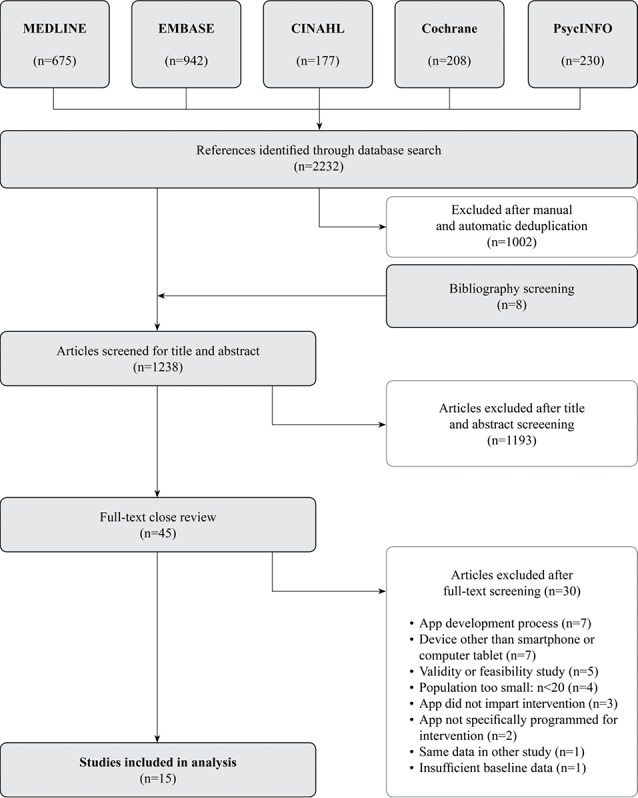
Study flow.

As shown in Figure 1, systematic literature search retrieved 2232 studies, which were reduced to 1258 after deduplication. After further manual deduplication, 1230 studies remained. After an additional bibliography screening of the relevant studies, 8 additional scientific studies were included, leading to 1238 studies. After title and abstract screening, 1193 articles were excluded. Finally, 45 full texts were reviewed closely using inclusion and exclusion criteria as well as criteria for the methodological quality, resulting in 15 eligible studies. The main reasons for exclusion are displayed in Figure 1.

### Study Overview

Characteristics of included studies are shown in Table 1 and in Multimedia Appendix 2. A total of 7 RCTs [21-27], 6 before-after studies [28-33], 1 controlled before-after study [34], and one retrospective data analysis [35] with a total of 1962 patients were reviewed. Of these, 11 studies were conducted in an out-clinic setting and 4 studies in an in-clinic setting. In addition, 8 of the studies were controlled (7 RCTs and 1 controlled before-after study); 1 was a 3-armed RCT. Moreover, 7 studies were single arm, 6 of which compared baseline to follow-up parameters (before and after), and 1 was a retrospective analysis of collected data [35]. Studies were published between 2015 and early 2018. Publication date of Blödt et al [21] differs from literature search because citation recommendation is dated for 2018, whereas the article was available online in 2017. The mean age of participants ranged between 12 and 68 years, and the follow-up period ranged between 0 and 28 days for in-clinic setting and 14 and 180 days for out-clinic setting. A total of 15 studies were conducted using a smartphone, 3 gave the possibility to use an app designed for multiple devices (smartphone, computer tablet, and computer [23,33,35]), and 2 studies were conducted solely with computer tablets [26,34]. A total of 12 apps were used for treatment of chronic pain; 2 interventions were used for management of singular acute pain [26,28] and 1 for recurrent (menstrual) pain [21]. Detailed information about each app used in the included studies can be found in Multimedia Appendix 3.

### Missing Data

One study did not report mean pain values but only mean change in pain values. Therefore, description of results but no graphic representation was possible [26]. Schatz et al [24] did not compare baseline to follow-up mean pain values, and consequently, the study could also not be included in the graphical representation. Authors of studies with missing data were contacted through email (corresponding email address on publication), but none of them responded. Huber et al [35] did not report the last day of use of the app in their intention-to-treat (ITT) analysis. Therefore, graphical representation was not possible for ITT analysis (missing x-axis value) but only for patients who completed the full observational time of this retrospective analysis.

### Effects of Apps on Pain

**Figure 2:**
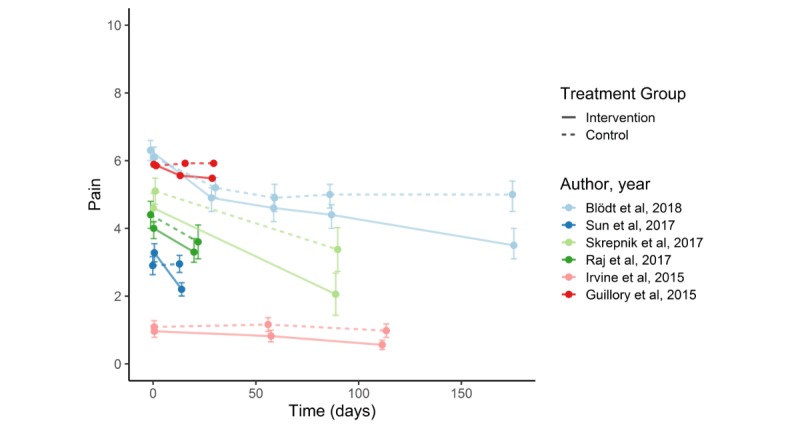
Improvement in pain over time in multiple armed studies. Pain scale (Numeric Rating Scale): 0-10.

**Figure 3:**
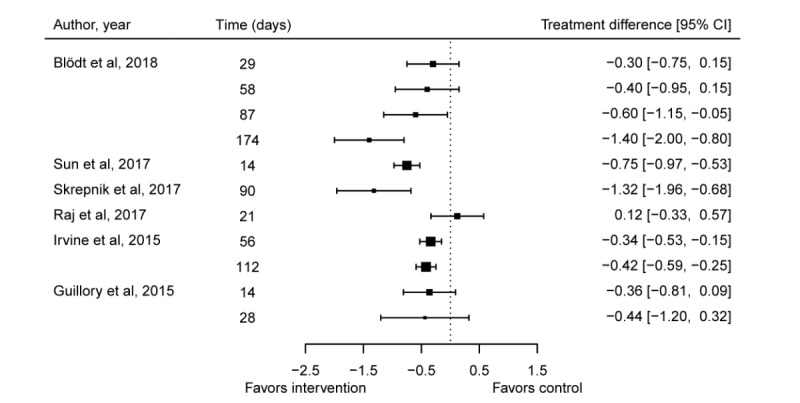
Forest plot of intervention effect on pain in multiple armed studies. Pain scale (Numeric Rating Scale): 0-10.

Figure 2 shows improvement in pain over time, and Figure 3 illustrates the effect size of the intervention in 6 controlled studies [21-23,25,27,34]. One study did not provide information about the standard deviation, and therefore, only mean pain values are shown in the graph [22]. In addition, 4 studies reported a significant improvement in pain in the group using the app [21,23,25,27]. In another study [21], patients showed no significant improvement at follow-up after 29 and 58 days. Skrepnik et al [25] reported only mean percentage change in walking pain; therefore, significance of mean change in pain was not possible to calculate. One study showed nonsignificant tendency toward improvement [22]; 1 study did not report improved pain in the intervention group [34].

**Figure 4:**
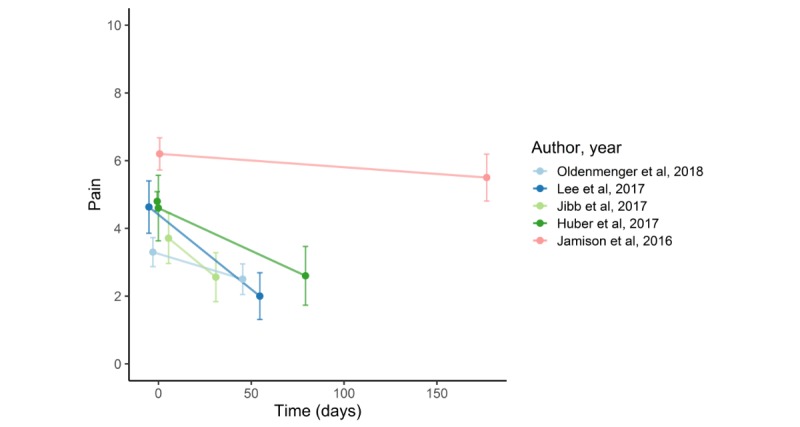
Decrease in pain in out-clinic before-after studies and in the retrospective data analysis. Pain scale (Numeric Rating Scale): 0-10.

**Figure 5:**
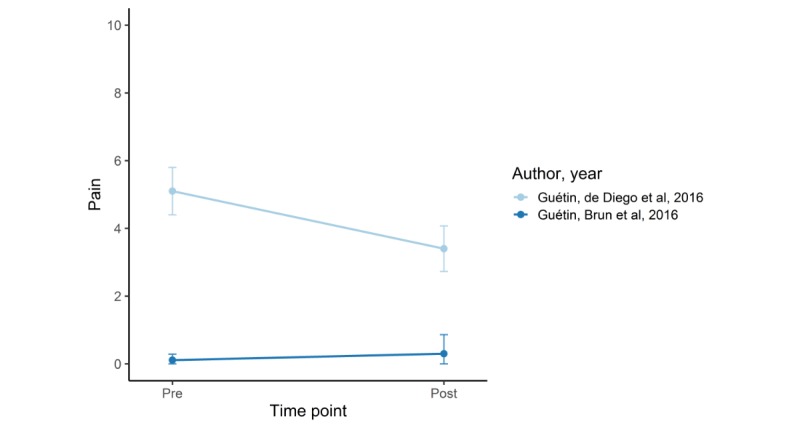
Decrease in pain in in-clinic before-after studies. Pain scale (Numeric Rating Scale): 0-10.

Figure 4 shows a mean decrease in pain over time in 5 single-arm studies [30-33,35]. All studies showed a significant decrease over time. Huber et al’s [35] ITT comparison of baseline pain to mean pain at the end of the observational time showed statistically significant decrease. Figure 5 shows a decrease in pain in 2 single-arm studies, with immediate postintervention measurements. One study showed a statistically significant decrease in chronic pain; the other showed no decrease in patients with acute pain [28,29]. Schatz et al [24] did not report about the severity of pain at baseline and follow-up. Therefore, we were not able to depict the results graphically. However, analysis based on daily pain diary data showed lower next-day pain in the intervention group compared with control group. Stinley et al [26] did not provide mean pain measurements; therefore, graphic depiction was also not possible. They reported that pain scores between groups did not differ.

**Table 1:** Baseline characteristics of all included studies.

Author, year	Study design	Device	Patients, N	Female, n (%)	Age, mean (SD)	Follow up (days)	Type of pain
Blödt et al, 2018 [21]	RCT^a^	S^b^	221	221 (100)	24 (3.6)	174^c^	Recurrent menstrual pain
Sun et al, 2017 [27]	RCT	S	46	14 (30.4)	67.5 (N/A^d^)	14	Chronic cancer
Skrepnik et al, 2017 [25]	RCT	S	211	106 (50.2)	62.6 (9.4)	90	Chronic walking pain
Raj et al, 2017 [34]	cBAS^e^	T^f^	214	103 (48.1)	60.1 (12.7)	21	Chronic cancer
Oldenmenger et al, 2017 [33]	BAS^g^	S/T/C^h^	84	44 (52.4)	59 (11)	42	Chronic cancer
Lee et al, 2017 [32]	BAS	S	23	10 (43.5)	28.1 (3)	56	Chronic neck pain
Jibb et al, 2017 [31]	BAS	S	40	17 (42.5)	14.2 (1.7)	28	Chronic cancer
Huber et al, 2017 [35]	RDA^i^	S/T/C	180	105 (58.3)	33.9 (10.9)	84	Chronic low back pain
Jamison et al, 2016 [30]	BAS	S	90	58 (64.4)	46.7 (12.9)	180	Chronic pain
Guétin, de Diego et al, 2016 [29]	BAS	S	53	42 (79.3)	47.4 (16.5)	—^j^	Chronic pain
Guétin, Brun et al, 2016 [28]	BAS	S	35	17 (48.6)	61.3 (11.6)	—	Acute pain before coronarography
Stinley et al, 2015 [26]	RCT	T	40	20 (50)	12.3 (2.9)	—	Acute needle stick pain
Schatz et al, 2015 [24]	RCT	S	46	27 (58.7)	13 (2.5)	112	Chronic pain in sickle cell disease
Irvine et al, 2015 [23]	RCT	S/T/C	597	358 (60)	N/A (N/A)	112	Chronic low back pain
Guillory et al, 2015 [22]	RCT	S	82	51 (75)	48.6 (11.6)	28	Chronic non cancer pain

^a^RCT: randomized controlled trial.

^b^S: smartphone.

^c^Duration of six menstruation cycles with a mean of 29 days.

^d^N/A: not available.

^e^cBAS: controlled before-after study.

^f^T: computer tablet

^g^BAS: before-after study.

^h^C: computer.

^i^RDA: retrospective data analysis.

^j^Not applicable.

### Effects of Apps on Other Outcomes and Information About Feasibility

Blödt et al [21] reported that patients using the app had pain for fewer days and needed less pain medication compared with the control group. In 2 studies [21,33], worst pain improved using the app but did not in another study [34]. Furthermore, statistically significant decreases were found in momentary pain, total pain interference (eg, general activity or mood), and pain catastrophizing in 1 study [30]. Anxiety decreased in patients using the app in 2 studies [28,29], whereas there was no reduction in another study [30]. Stinley et al [26] reported only a decrease in anxiety in the subgroup of patients with high anxiety levels. Guillory et al [22] reported statistically significant improvements in pain interference with general activities, pain interference with sleep, pain interference in relation with others, and positive affect for app users compared with the control group during the intervention period. However, 1 week after the intervention period, sleep pain and positive affect were not significant any more. In another study, statistically significant improvements in functionality, well-being, productivity, and presenteeism at work were reported for the treatment group compared with the control group [23]. One study found that age, ethnicity, and gender did not influence compliance or satisfaction with the app [30].

All studies evaluating changes in quality of life revealed a statistically significant improvement in the groups using an app [23,27,31,32]. In addition, 8 studies reported high satisfaction with the ease of use of the app [21,23,25,27-29,33,36], and 3 studies stated good feasibility of app use with high diary completion rates [22,27,31]. Further details about the additional outcomes can be found in Multimedia Appendix 4.

### Methodological Quality of the Randomized Controlled Trials

Multimedia Appendix 5 shows the risks of biases of all included RCTs using the corresponding SIGN checklist [20]. Furthermore, 1 RCT was of high quality [24], and 6 RCTs [21-23,25-27] were of acceptable quality. The studies of acceptable quality had a deficiency in at least one of the following areas: description of random allocation (checklist item 1.2), concealment (1.3), comparability at baseline (1.5), missing ITT analysis (1.9), or missing multisite comparisons (1.10).

## Discussion

### Principal Findings

The main objective of this review was to evaluate the efficacy of apps on smartphones or tablets for the management of patients with pain. The results of 7 RCTs, 6 before-after studies, 1 controlled before-after study, and 1 retrospective data analysis were included in this review. The majority of the original studies reported beneficial effects of the use of a pain app. Severity of pain decreased in most studies in patients using an app compared with those not using an app. Other outcomes, such as *worst pain* or *quality of life*, showed improvements in patients using an app. Due to heterogeneity between the original studies—patient characteristics, app content, and study setting—a synthesis of the results by statistical methods was not performed.

### Comparison With Existing Literature

To the best of our knowledge, this is the first systematic review about the efficacy of smartphone or computer tablet apps for the management of patients with pain. There are published systematic reviews that study the quality of the content (eg, evidence-based interventions or inclusion of health care professionals in the development process) and ease of use of commercially available apps for pain management [10-15,17]. None of the reviews that were reported assessed the overall effectiveness of apps as tools for managing pain in patients.

Authors of 3 reviews [10-12] criticized that the apps were not comprehensive in terms of pain self-management and that health care professionals and patients were scarcely involved in the development of the app. Furthermore, they reported a lack of scientific evidence in app content. Machado et al [13] examined the quality of apps for low back pain. Although 58 out of 61 apps included some type of intervention (eg, information for strengthening and/or stretching exercises and yoga, which was listed in the National Institute for Health and Care Excellence guidelines for low back pain [37]), most of the assessed apps lacked evidence and were of poor quality. Bhattarai et al [14] evaluated smartphone apps for self-management in patients with arthritic pain in their systematic review. Out of 373 assessed apps, only 4 met the authors’ inclusion criteria, and 3 of them did not fulfill the minimal usability criteria. Portelli and Eldred [15] reviewed the degree to which apps adhere to evidence-based practices in psychological research for pain management. Integrated theoretical reference to cognitive behavioral therapy (CBT) principles was only present in 6 out of 195 apps investigated.

Other studies not meeting our inclusion criteria (eg, use of other devices or use of apps not specifically made for intervention) showed results similar to the studies included in our analysis. In the study of Kristjánsdóttir et al [38], patients used early smartphone models to fill in pain diaries on the internet and to communicate with the therapist through SMS in a 4-week intervention trial. As a result, catastrophizing of pain was reduced in the intervention compared with the control group after 4 weeks and 5 months, but benefits were no longer evident after an 11-month follow-up assessment [39]. Somers et al [40] demonstrated that remote pain-coping skills training delivered via videoconferencing on a computer tablet is feasible and effective in decreasing pain, reducing psychological distress, and pain catastrophizing. Basch et al [41] demonstrated that access to a Web-based collection of information about symptoms at home during chemotherapy with alerts to treating staff is beneficial for patients. The intervention group had better quality of life, less emergency room admissions, and longer chemotherapy than the control group.

Reviews about the effects of apps in other medical fields showed reduction in anxiety [42] and depressive symptoms [43] as well as improvement in asthma control and lung function [5]. Another study reported the use of a medication app, which improved medication adherence and lowered the rate or number of missed medications [44]. In patients with diabetes, apps shortened times for clinical personnel to review glucose diaries compared with the traditional personal glucose diaries [7] and have the potential of improving glucose control in type 1 and 2 diabetes [7,8], as well as increasing adherence to treatment in patients older than 60 years [45]. Another app was effective in promoting physical activity measured as steps per day after 8 weeks [46].

One study reported no improvement in systolic blood pressure with app use, although there was a small improvement in medication adherence [47]. Hurkmans et al [48] showed that addition of an app in a weight loss program did not improve dietary patterns or physical activity.

### Comment on Results

For the sake of overview, we depicted acute and chronic pain outcome studies in the same figures. However, acute pain and chronic pain are two different entities; therefore, direct comparison is difficult, and this should be considered when interpreting the results. Furthermore, part of our results showing no effect of app use on pain management can be explained. The app examined by Raj et al [34] was a clinical decision support tool for physicians treating patients with cancer pain to evaluate improvement in pain control and to suggest treatment changes in opioid prescription (developed by mathematical algorithms). However, physicians were free to use or ignore the treatment suggestions provided by the app. Effectively, there was no statistically significant difference in either new prescription of or change in opioid medication in the intervention group compared with the control group. Guétin et al [28] did not report a decrease in pain during coronarography, which is not surprising because of the absence of pain in most patients before the procedure. Stinley et al [26] investigated pain and anxiety during venepuncture in children. The intervention group generated a mandala (defined by the authors as a drawing in which artists create a design using a circular outline [26]) on a computer tablet, and the control group received standard care treatment. The absolute pain levels were not reported; therefore, understanding why the intervention did not improve pain is difficult.

### Limitations and Strengths

The main limitation of our review is the heterogeneity of the included studies for the synthesis of study results. On the other hand, this enables existing data to be represented in general without selection bias. Furthermore, apps are a new element in telehealth and their development is still progressing. Indeed, apps were only made available for a wide population in 2008 and 2009, when the Apple’s App store and Google Play for Android, respectively, were introduced. Our strength is that this review is based on the PRISMA guidelines [18] and that the SIGN checklists [20] were used to critically appraise the included studies. The heterogeneity between the studies regarding the eligibility criteria, interventions, apps, and outcome selection prevented a meta-analysis. We attempted to compensate for this limitation by supplying a well-balanced, detailed, and qualitative review of the studies included.

### Implications

In the management of patients with pain, apps can provide patients a wide range of features such as pain diary, educational features, reminders, treatment recommendation aspects, and direct communication with health care personnel in a single mobile app. Furthermore, intelligent systems such as chatbots or virtual assistants are already part of daily life for many people. These new technologies should be introduced into telehealth in the near future, and their testing for validity and usability is crucial. Correspondingly, there is a need for more high-quality studies for the evaluation of the efficacy of these new instruments.

Furthermore, our review showed that a standardized assessment of pain is lacking in the included studies, and therefore, it would be desirable that the scientific community agrees on a standardized protocol for pain assessment. In addition, there is a need for detailed reporting of the structure, data assessment, and functions of the apps and studies to investigate the elements of the apps or additional devices or tools that may improve usability and help patients in pain management. This information would strengthen future studies and allow researchers to synthesize the results of different studies.

### Conclusions

Apps for pain management may be beneficial for patients, particularly in an out-clinic setting. Studies have shown that pain apps are workable and well liked by patients and health care professionals. There is no doubt that in the near future, mobile technologies will develop further. Medicine could profit from this development as our results indicate, but there is a need for more scientific inputs. It is desirable to know which elements of apps or additional devices or tools may improve usability and help patients in pain management.

## Appendix

Multimedia Appendix 1 Search strategy for Ovid MEDLINE(R).

Multimedia Appendix 2 Baseline characteristics of all included studies.

Multimedia Appendix 3 Detailed information of apps used in the included studies.

Multimedia Appendix 4 Additional outcomes of interest.

Multimedia Appendix 5 Scottish Intercollegiate Guidelines Network (SIGN) checklist for randomized controlled trials.

## Abbreviations

ITT: intention-to-treat
PRISMA: Preferred Reporting Items for Systematic Reviews and Meta-Analyses
NRS: Numeric Rating Scale
RCT: randomized controlled trial
SIGN: Scottish Intercollegiate Guidelines Network
SMS: short message service
VAS: Visual Analog Scale
