# Supplementary material for: Benefits of Mobile Apps in Pain Management: Systematic Review
Source: JMIR Mhealth Uhealth. 2018 Oct 22;6(10):e11231. doi: 10.2196/11231 (PMC6231845; doi:10.2196/11231)
Supplement: Multimedia Appendix 1 [file mhealth_v6i10e11231_app1.pdf]

Multimedia Appendix 1: Search strategy for Ovid MEDLINE(R).

	**Searches**	**Results**
1	("27713649" or "25565585" or "26769149" or "28811270" or "28528981" or "24999983" or "24921074" or "25138438" or "12237213" or "27898460" or "11275385" or "28423223" or "23291270" or "23538392" or "25370138" or "29061558" or "20580526" or "14736583" or "28337948" or "27089351" or "27583140" or "24422990" or "22133450" or "24535055" or "26507916" or "28570436" or "24604571").ui.	31
2	exp Pain/ or (pain or headache or backache).ti,ab. 772082	772’082
3	exp cell phones/ or exp Computers, Handheld/ or exp Mobile Applications/ or exp Telemedicine/ or (smartphone* or ipad* or " Personal Digital Assistant*" or pda* or palmtop*).ti,ab. or ((mobile or portable) adj3 (phone* or device*)).ti,ab. or ((internet or web or electronic) adj3 diar*).ti,ab. or (app or apps).ti. or ((mobile or e-health or ehealth or m-health or mhealth or electronic or digital or web or internet) adj3 application*).ti,ab.	73’083
4	2 and 3	1’605
5	exp Pain Management/ or (pain adj4 (manag* or monitor* or scal* or scor* or assess* or intensit* or diar*)).ti,ab. or (self adj1 (manag* or monitor* or report*)).ti,ab.	284’749
6	2 and 3 and 5	689
7	limit 6 to (english or german)	675
8	1 not 7	1
