# Supplementary material for: Benefits of Mobile Apps in Pain Management: Systematic Review
Source: JMIR Mhealth Uhealth. 2018 Oct 22;6(10):e11231. doi: 10.2196/11231 (PMC6231845; doi:10.2196/11231)
Supplement: Multimedia Appendix 2 [file mhealth_v6i10e11231_app2.pdf]

Multimedia Appendix 2: Baseline characteristics of all included studies.

Author, Year	Study design	Groups, n	Device	Setting	Patients, N	Female, n (%)	Mean age (SD)	Follow up, days	Type of pain	Type of App
Blödt et al, 2018 [21]	RCT[a]	2	S[b]	out-clinic	221	221 (100)	24 (3.6)	174[c]	recurrent menstrual pain	pain diary
Sun et al, 2017 [27]	RCT	2	S	out-clinic	46	14 (30.4)	67.5 (N/A[d])	14	chronic cancer	pain scale
Skrepnik et al, 2017 [25]	RCT	2	S	out-clinic	211	106 (50.2)	62.6 (9.4)	90	chronic walking pain	pain scale
Raj et al, 2017 [34]	cBAS[e]	2	T[f]	in-clinic	214	103 (48.1)	60.1 (12.7)	21	chronic cancer	pain scale
Oldenmenger et al, 2018 [33]	BAS[g]	1	S/T/C[h]	out-clinic	84	44 (52.4)	59 (11)	42	chronic cancer	pain diary
Lee et al, 2017 [32]	BAS	1	S	out-clinic	23	10 (43.5)	28.1 (3)	56	chronic neck pain	pain scale
Jibb et al, 2017 [31]	BAS	1	S	out-clinic	40	17 (42.5)	14.2 (1.7)	28	chronic cancer	pain scale
Huber et al, 2017 [35]	RDA[i]	1	S/T/C	out-clinic	180	105 (58.3)	33.9 (10.9)	84	chronic low back pain	pain diary
Jamison et al, 2016 [30]	BAS	1	S	out-clinic	90	58 (64.4)	46.7 (12.9)	180	chronic pain	pain scale
Guétin, de Diego et al, 2016 [29]	BAS	1	S	in-clinic	53	42 (79.3)	47.4 (16.5)	n.a.[j]	chronic pain	receptive music intervention
Guétin, Brun et al, 2016 [28]	BAS	1	S	in-clinic	35	17 (48.6)	61.3 (11.6)	n.a.	acute pain before coronarography	receptive music intervention
Stinley et al, 2015 [26]	RCT	2	T	in-clinic	40	20 (50)	12.3 (2.9)	n.a.	acute needle stick pain	distraction while intervention
Schatz et al, 2015 [24]	RCT	2	S	out-clinic	46	27 (58.7)	13 (2.5)	112	chronic pain in sickle cell disease	pain diary
Irvine et al, 2015 [23]	RCT	3	S/T/C	out-clinic	597	358 (60)	N/A (N/A)	112	chronic low back pain	pain scale
Guillory et al, 2015 [22]	RCT	2	S	out-clinic	82	51 (75)	48.6 (11.6)	28	chronic non cancer pain	pain scale

[a]RCT: Randomized controlled trial.
[b]S: Smartphone.
[c]: Duration of six menstruation cycles with a mean of 29 days.
[d]N/A: Not available.
[e]cBAS: Controlled before-after study.
[f]T: Computer tablet
[g]BAS: Before-after study.
[h]C: Computer.
[i]RDA: Retrospective data analysis.
[j]n.a.: Not applicable.

References:

21. Blödt S, Pach D, Eisenhart-Rothe SV, Lotz F, Roll S, Icke K, Witt CM. Effectiveness of app-based self-acupressure for women with menstrual pain compared to usual care: a randomized pragmatic trial. Am J Obstet Gynecol 2018 Feb; 218(2):227.e1-227.e9
22. Guillory J, Chang P, Henderson CR, Shengelia R, Lama S, Warmington M, Jowza M, Waldman S, Gay G, Reid MC. Piloting a Text Message-based Social Support Intervention for Patients With Chronic Pain: Establishing Feasibility and Preliminary Efficacy. Clin J Pain 2015 Jun; 31(6):548-56
23. rvine AB, Russell H, Manocchia M, Mino DE, Cox GT, Morgan R, Gau JM, Birney AJ, Ary DV. Mobile-Web app to self-manage low back pain: randomized controlled trial. J Med Internet Res 2015 Jan 02; 17(1):e1
24. Schatz J, Schlenz AM, McClellan CB, Puffer ES, Hardy S, Pfeiffer M, Roberts CW. Changes in coping, pain, and activity after cognitive-behavioral training: a randomized clinical trial for pediatric sickle cell disease using smartphones. Clin J Pain 2015 Jun; 31(6):536-47
25. Skrepnik N, Spitzer A, Altman R, Hoekstra J, Stewart J, Toselli R. Assessing the Impact of a Novel Smartphone Application Compared With Standard Follow-Up on Mobility of Patients With Knee Osteoarthritis Following Treatment With Hylan G-F 20: A Randomized Controlled Trial. JMIR Mhealth Uhealth 2017 May 09; 5(5):e64
26. Stinley N, Norris D, Hinds P. Creating Mandalas for the Management of Acute Pain Symptoms in Pediatric Patients. Art Therapy 2015 Jun 22; 32(2):46-53
27. Sun Y, Jiang F, Gu JJ, Wang YK, Hua H, Li J, Cheng Z, Liao Z, Huang Q, Hu W, Ding G. Development and Testing of an Intelligent Pain Management System (IPMS) on Mobile Phones Through a Randomized Trial Among Chinese Cancer Patients: A New Approach in Cancer Pain Management. JMIR Mhealth Uhealth 2017 Jul 25; 5(7):e108
28. Guétin S, Brun L, Deniaud M, Clerc J, Thayer JF, Koenig J. Smartphone-based Music Listening to Reduce Pain and Anxiety Before Coronarography: A Focus on Sex Differences. Altern Ther Health Med 2016 Jul; 22(4):60-3
29. Guétin S, Diego E, Mohy F, Adolphe C, Hoareau G, Touchon J, Thayer Jf, Koenig J. A patient-controlled, smartphone-based music intervention to reduce pain—A multi-center observational study of patients with chronic pain. European Journal of Integrative Medicine 2016 Jun; 8(3):182-187
30. Jamison RN, Mei A, Ross EL. Longitudinal trial of a smartphone pain application for chronic pain patients: Predictors of compliance and satisfaction. J Telemed Telecare 2018 Feb; 24(2):93-100
31. Jibb LA, Stevens BJ, Nathan PC, Seto E, Cafazzo JA, Johnston DL, Hum V, Stinson JN. Implementation and preliminary effectiveness of a real-time pain management smartphone app for adolescents with cancer: A multicenter pilot clinical study. Pediatr Blood Cancer 2017 Oct; 64(10)
32. Lee M, Lee SH, Kim T, Yoo H, Kim SH, Suh D, Son J, Yoon B. Feasibility of a Smartphone-Based Exercise Program for Office Workers With Neck Pain: An Individualized Approach Using a Self-Classification Algorithm. Arch Phys Med Rehabil 2017 Dec; 98(1):80-87
33. Oldenmenger WH, Baan MAG, van der Rijt CCD. Development and feasibility of a web application to monitor patients' cancer-related pain. Support Care Cancer 2018 Feb; 26(2):635-642
34. Raj SX, Brunelli C, Klepstad P, Kaasa S. COMBAT study - Computer based assessment and treatment - A clinical trial evaluating impact of a computerized clinical decision support tool on pain in cancer patients. Scand J Pain 2017 Oct; 17:99-106
35. Huber S, Priebe JA, Baumann K, Plidschun A, Schiessl C, Tölle TR. Treatment of Low Back Pain with a Digital Multidisciplinary Pain Treatment App: Short-Term Results. JMIR Rehabil Assist Technol 2017 Dec 04; 4(2):e11
