# Supplementary material for: Benefits of Mobile Apps in Pain Management: Systematic Review
Source: JMIR Mhealth Uhealth. 2018 Oct 22;6(10):e11231. doi: 10.2196/11231 (PMC6231845; doi:10.2196/11231)
Supplement: Multimedia Appendix 3 [file mhealth_v6i10e11231_app3.pdf]

Multimedia Appendix 3. Detailed information of apps used in the included studies.

**Author, year**	**Device**	**Operating system**	**Name of app, availability**	**App content**
Blödt et al, 2018 [21]	S[a]	Android, iOS[b]	AKUD, publicly available	− Questionnaires;<br>− Diary;<br>− Visualization of the menstrual cycle;<br>− Additional features in intervention group: acupressure-specific features (acupressure procedure explanation, drawings, videos, photos, and a timing function); reminder function (switch off possible)
Sun et al, 2017 [27]	S	Android	N/A[c], N/A	− Life quality self-evaluation;<br>− Cancer pain self-evaluation;<br>− Real-time messaging (with doctor);<br>− Standard medication (medication reminder)
Skrepnik et al, 2017 [25]	S	iOS	OA GO, N/A	− Motivational messages;<br>− Requesting patient entering pain and mood data once a day;<br>− Daily step goals;<br>− The OA GO app combined continuously retrieved data from the wearable activity monitor with data entered by the patient to display the patient’s daily step count, calories burned, and sleep. Daily and monthly cumulative activity trends were available for the patient to review.
Raj et al, 2017 [34]	T[d]	iOS	COMBAT system, not publicly available	− Computer-based collection of PROMs[e];<br>− Immediate wireless transfer of PROMs to the oncologist computer at point-of-care;<br>− Computerized clinical decision support systems designed for cancer pain management
Oldenmenger et al, 2018 [33]	S/T/C[f]	Android, iOS	Gezondheidsmeter, publicly available	− Pain diary for monitoring, medication side effects, and analgesics intake;<br>− Pain education module: information about pain, use of medication and side effects, and concerns and misconceptions about pain and pain treatment;<br>− eConsult module for communication between the patient and the health professional; data storage on safe server; automatic alerts to nurse specialist.
Lee et al, 2017 [32]	S	Android, iOS	N/A, N/A	− Self-classification algorithm for patients to self-classify their type of neck pain;<br>− Corresponding exercise program depending on the type of neck pain consisting of 10 postures was generated automatically by the app.<br>− Adherence to the exercise program was measured by the number of exercise sessions completed, expressed as a percentage of the 24 possible sessions during the 8 weeks (eg, adherence rate) and the mean duration per exercise session. Both of these were calculated

Author, year	Device	Operating system	Name of app, availability	App content
				automatically by the app when the participants logged into the app and pressed the *finish* button after completing the exercise.
Jibb et al, 2017 [31]	S	iOS	Pain Squad+, publicly available	− Pain assessments twice daily (repeated audible notifications); 22-item questionnaire (intensity of pain, its duration and location, pain causes, treatment strategies used, perceived control in managing pain, affective impact of pain, and its interference with activities of daily living);<br>− Ad hoc pain assessments using a truncated 8-item questionnaire (pain intensity, control in managing pain, and key pain interference items [eg, interference with sleep]) anytime between the morning and evening assessments;<br>− After pain reporting, patients received real-time self-management recommendations from the app according to a standardized algorithm (pharmacological, psychological, or physical); 1 hour later, reassessing of pain and additional advice as appropriate.;<br>− Email alerts if an adolescent reported pain of more than 3/10 on three consecutive occasions. The nurse then contacts the adolescent and/or their primary medical team to discuss the case and initiate provider-driven intervention such as changes in medications;<br>− To encourage engagement with Pain Squad+, the app is *gamified*
Huber et al, 2017 [35]	S/T/C	Android, iOS, native Web solutions	Kaia, publicly available	− Data of self-test and in-APP diaries (pain and sleep);<br>− Back pain-specific education (over 30 different educational units)[g];<br>− Physiotherapy (out of 145 exercises)[f];<br>− Mindfulness techniques (breathing techniques, body scan, visualization, and progressive muscle relaxation)[g];<br>− Chat function with coach (physiotherapist or sport scientist)
Jamison et al, 2016 [30]	S	Android, iOS	N/A, N/A	− Demographic and contact information;<br>− Chronic pain assessment;<br>− 5-item daily assessments with push notification reminders;<br>− Personalized goal setting;<br>− Topics of interest with psychological and medical pain management strategies;<br>− Saved line graphs from the daily assessments that were placed on the patients' medical record;<br>− Participants were also supplied a Fitbit to track their daily activity (Fitbit Zip, San Francisco, California, USA).

Author, year	Device	Operating system	Name of app, availability	App content
Guetin et al, 2016 [29]	S	N/A	Music Care, publicly available	− Receptive music intervention; standardized musical sequence of 20 min in length (standardized music session of 20 min broken down into several phases, which gradually help the patient to relax according to the so-called *U* sequence);<br>− The music has solely been recorded for use within the application with a variety of 20 different musical styles.
Guetin et al, 2016 [28]	S	N/A	Music Care, publicly available	− Receptive music intervention; standardized musical sequence of 20 min in length (standardized music session of 20 min broken down into several phases, which gradually help the patient to relax according to the so-called *U* sequence);<br>− The music has solely been recorded for use within the application with a variety of 20 different musical styles.
Stinley et al, 2015 [26]	T	iOS	Sketchbook Pro 2.8 by Autodesk, publicly available	− Mandala template created by author to color or draw anything inside or outside the circle.
Schatz et al, 2015 [24]	S	Windows Mobile, Android	N/A, N/A	− Daily diary application (pain, participation in daily activities, and coping skill use);<br>− Coping skills program; skill practice or use application to access audio files for deep breathing, progressive muscle relaxation, and guided imagery.
Irvine et al, 2015 [23]	S/T/C	Android, iOS, native Web solutions	FitBack, N/A	− Mobile Web app that provides nonspecific low back pain education and behavioral strategies to manage current pain and prevent future pain episodes using a self-tailored, cognitive-behavioral approach allowing users to develop and support self-efficacy;<br>− Using pain and activity self-monitoring, gain-framed text and video messages;<br>− Tracking of daily pain management activities through an *activity picker* populated with pain self-care activities in 4 categories (rest and relief, mindfulness, general fitness, and back pain-specific stretching and strength exercises);<br>− Providing users with graphs showing trends of pain level.
Guillory et al, 2015 [22]	S	Android, iOS	N/A, N/A	− Pain tracking app (pain and pain interference data)

[a]S: Smartphone.

[b]iOS: iPhone operating system.

[c]N/A: Not available.

[d]T: Tablet.

[e]PROMs: Patient-reported outcome measures.

[f]C: Computer.

[g]Content for an individual patient is compiled and updated from day to day.

References:

21. Blödt S, Pach D, Eisenhart-Rothe SV, Lotz F, Roll S, Icke K, Witt CM. Effectiveness of app-based self-acupressure for women with menstrual pain compared to usual care: a randomized pragmatic trial. Am J Obstet Gynecol 2018 Feb; 218(2):227.e1-227.e9
22. Guillory J, Chang P, Henderson CR, Shengelia R, Lama S, Warmington M, Jowza M, Waldman S, Gay G, Reid MC. Piloting a Text Message-based Social Support Intervention for Patients With Chronic Pain: Establishing Feasibility and Preliminary Efficacy. Clin J Pain 2015 Jun; 31(6):548-56
23. rvine AB, Russell H, Manocchia M, Mino DE, Cox GT, Morgan R, Gau JM, Birney AJ, Ary DV. Mobile-Web app to self-manage low back pain: randomized controlled trial. J Med Internet Res 2015 Jan 02; 17(1):e1
24. Schatz J, Schlenz AM, McClellan CB, Puffer ES, Hardy S, Pfeiffer M, Roberts CW. Changes in coping, pain, and activity after cognitive-behavioral training: a randomized clinical trial for pediatric sickle cell disease using smartphones. Clin J Pain 2015 Jun; 31(6):536-47
25. Skrepnik N, Spitzer A, Altman R, Hoekstra J, Stewart J, Toselli R. Assessing the Impact of a Novel Smartphone Application Compared With Standard Follow-Up on Mobility of Patients With Knee Osteoarthritis Following Treatment With Hylan G-F 20: A Randomized Controlled Trial. JMIR Mhealth Uhealth 2017 May 09; 5(5):e64
26. Stinley N, Norris D, Hinds P. Creating Mandalas for the Management of Acute Pain Symptoms in Pediatric Patients. Art Therapy 2015 Jun 22; 32(2):46-53
27. Sun Y, Jiang F, Gu JJ, Wang YK, Hua H, Li J, Cheng Z, Liao Z, Huang Q, Hu W, Ding G. Development and Testing of an Intelligent Pain Management System (IPMS) on Mobile Phones Through a Randomized Trial Among Chinese Cancer Patients: A New Approach in Cancer Pain Management. JMIR Mhealth Uhealth 2017 Jul 25; 5(7):e108
28. Guétin S, Brun L, Deniaud M, Clerc J, Thayer JF, Koenig J. Smartphone-based Music Listening to Reduce Pain and Anxiety Before Coronarography: A Focus on Sex Differences. Altern Ther Health Med 2016 Jul; 22(4):60-3
29. Guétin S, Diego E, Mohy F, Adolphe C, Hoareau G, Touchon J, Thayer Jf, Koenig J. A patient-controlled, smartphone-based music intervention to reduce pain—A multi-center observational study of patients with chronic pain. European Journal of Integrative Medicine 2016 Jun; 8(3):182-187
30. Jamison RN, Mei A, Ross EL. Longitudinal trial of a smartphone pain application for chronic pain patients: Predictors of compliance and satisfaction. J Telemed Telecare 2018 Feb; 24(2):93-100
31. Jibb LA, Stevens BJ, Nathan PC, Seto E, Cafazzo JA, Johnston DL, Hum V, Stinson JN. Implementation and preliminary effectiveness of a real-time pain management smartphone app for adolescents with cancer: A multicenter pilot clinical study. Pediatr Blood Cancer 2017 Oct; 64(10)
32. Lee M, Lee SH, Kim T, Yoo H, Kim SH, Suh D, Son J, Yoon B. Feasibility of a Smartphone-Based Exercise Program for Office Workers With Neck Pain: An Individualized Approach Using a Self-Classification Algorithm. Arch Phys Med Rehabil 2017 Dec; 98(1):80-87
33. Oldenmenger WH, Baan MAG, van der Rijt CCD. Development and feasibility of a web application to monitor patients' cancer-related pain. Support Care Cancer 2018 Feb; 26(2):635-642
34. Raj SX, Brunelli C, Klepstad P, Kaasa S. COMBAT study - Computer based assessment and treatment - A clinical trial evaluating impact of a computerized clinical decision support tool on pain in cancer patients. Scand J Pain 2017 Oct; 17:99-106
35. Huber S, Priebe JA, Baumann K, Plidschun A, Schiessl C, Tölle TR. Treatment of Low Back Pain with a Digital Multidisciplinary Pain Treatment App: Short-Term Results. JMIR Rehabil Assist Technol 2017 Dec 04; 4(2):e11
