# Supplementary material for: Benefits of Mobile Apps in Pain Management: Systematic Review
Source: JMIR Mhealth Uhealth. 2018 Oct 22;6(10):e11231. doi: 10.2196/11231 (PMC6231845; doi:10.2196/11231)
Supplement: Multimedia Appendix 4 [file mhealth_v6i10e11231_app4.pdf]

## Multimedia Appendix 4. Additional outcomes of interest.

| Author, year | Primary/secondary/applicability (p/s/a)[a]: outcome* | Intervention-control-effect |
|---|---|---|
| Blödt et al, 2018 [21] | **s:** worst pain; number of days with pain; women with medication intake; number of days with pain medication; general improvement in menstrual pain; responder rate; sick leave days; body efficiency expectation; | **s:** sig.[b] improvement; most at sixth cycle in: worst pain; number of days with pain; women with medication intake; number of days with pain medication; general improvement in menstrual pain; responder rate; no improvement in: sick leave days; body efficiency expectation; |
| | **a:** satisfaction; practice time | **a:** medium (to high) satisfaction with intervention; practice time shortened progressively during study time |
| Sun et al, 2017 [27] | **p:** feasibility; | **a/p:** feasibility showed high compliance rate with no difference between weekdays and weekends; |
| | **s:** acquired pain management knowledge; changes in quality of life; | **s:** sig. improvement intervention group to control group in pain management knowledge and quality of life; |
| | **a:** user satisfaction with app | **a:** 36% and 64% of patients liked it and very much liked it, respectively; patients also rated the app to be convenient; helpful for pain management; good technical support and good consultant and training course. Prompt response for help was rated high. |
| Skrepnik et al, 2017 [25] | **p:** mean chance from baseline to day 90 in mobility as measured by steps per day; | **p:** sig. improvement in mobility; |
| | **s:** PAM[c]-13 questionnaire score: percentage change in sleep: VAMS[e]; | **s:** PAM-13 improved in both groups sig. (no group difference): n.s.[d] change in and between groups in sleep and VAMS; |
| | **a:** patient and physician satisfaction | **a:** greater number of patients and physicians reported that they would use/recommend the devices than patients or physicians who would only somewhat likely or not use/recommend it |
| Raj et al, 2017 [34] | **p:** worst pain intensity in last 24 hours: prescribed opioid doses | **p:** no sig. difference in groups |
| Oldenmenger et al, 2018 [33] | **p/a:** feasibility; | **p/a:** primary goal not met but patient used app diary frequently; |
| | **s:** worst pain; | **s:** worst pain decreased sig. and more than mean pain; |
| | **a:** patients opinion about the app | **a:** patients found the time to fill in diary data to be satisfying; patients were very positive about the app; only minor technical difficulties occurred |
| Lee et al, 2017 [32] | **p:** functional disability measured with NDI[f]; | **p:** sig. and clinical meaningful lower NDI; |
| | **s:** quality of life measured with 36-item short-form health survey (SF-36): Fear avoidance measured with FABQ[g]: Cervical Range Of Motion ROM[h]; | **s:** sig. improvement in physical functioning; bodily pain; general health; vitality and physical component scores (all part of SF-36); FABQ: fear avoidance and physical activity and work subscale scores decreased but n.s.; increased ROM in all directions but n.s.; |
| | **a:** patient satisfaction: adherence | **a:** medium to high satisfaction; high adherence |

| Author, year | Primary/secondary/applicability (p/s/a)[a]: outcome* | Intervention-control-effect |
|---|---|---|
| Jibb et al, 2017 [31] | **p/a:** proportions of eligible patients: proportions of participants retained in study; intervention fidelity; proportion of outcome assessments completion; adherence; acceptability; | **p/a:** 77% participation of eligible patients; 95% of participants retained; fidelity was high; main problem was service disconnection and phone not in proximity; outcome completion was very high (>95%); participants perceived themselves to have moderate capacity to control pain; |
| | **s:** pain intensity: pain interference; life quality (Pediatric Quality of Life Inventory); self-efficacy (General Self-Efficacy-Sherer Scale-GSE-Sherer Scale) | **s:** all pain items, but current pain improved sig.; quality of life improved in total score sig.; self-efficacy did not improve |
| Huber et al, 2017 [35] | **p:** comparison of baseline to last day of use pain; difference in pain development between 12-week program completers and noncompleters; | **p:** sig. change in mean pain from baseline to last day of use; better pain outcomes in completers compared with non-completers with a medium-to-large effect; |
| | **s:** development of pain levels over time (three follow-up measures after 4, 8, and 12 weeks of use) | **s:** decrease in NRS levels was larger over time |
| Jamison et al, 2016 [30] | **p:** other elements of the Brief Pain Inventory; Pain Catastrophizing Scale; PDI[i]; HADS[j]; average steps per day (Fitbit track); | **p:** sig. decrease found in momentary pain, total pain interference and pain catastrophizing; no sig. changes in pain relief, least pain, PDI, HADS, and average steps per day; |
| | **a:** feasibility and tolerability of the pain app (number and frequency of daily assessments and how many subjects continued to use the app at the completion of the trial | **a:** 91% subjects submitted daily reports; 55.6% completed the 6-month evaluation; satisfaction questionnaire showed that the app was easy to use, send reports, and navigate, daily prompts were not bothersome, patients were willing to use the program every day (sig. less willing patients at 6 months compared with 3 months), patients also found the app appealing, daily reports were seen as useful; patients thought the app to be medium helpful to cope with pain; patients with more daily assessments were more satisfied with the app. |
| Guétin et al, 2016 [29] | **p:** difference between responders and nonresponders; | **p:** no difference in responders in age, occupational status, job description, study site of enrollment, diagnosis, musical program chosen, presession anxiety, presession rating of pain intensity, musical preferences, and experiences. Responders were more likely to not play an instrument; |
| | **s:** assessment of anxiety; | **s:** sig. reduction of anxiety after intervention; |
| | **a:** satisfaction with the intervention | **a:** satisfaction was high but did not improve sig. from first to last (seventh) session. |
| Guétin et al, 2016 [28] | **p:** anxiety; | **p:** sig. reduction in anxiety post intervention; |
| | **a:** satisfaction | **a:** majority of participants were satisfied or very satisfied |
| Stinley et al, 2015 [26] | **p:** heart rate; blood oxygen saturation; anxiety (Fears Rating Scale) | **p:** no difference in heart rate change between groups; blood oxygen did not differ between groups; sig. fewer treatment group participants showed stress behaviors during needle stick procedure; trend but no significance in anxiety between groups but within the treatment group compared with baseline; additional statistics showed that the high anxiety subgroup had sig. |

| Author, year | Primary/secondary/applicability (p/s/a)[a]: outcome* | Intervention-control-effect |
|---|---|---|
| | | higher decrease in heart rate compared with control group and that anxiety decreased sig. more |
| Schatz et al, 2015 [24] | **p:** CSQ[k], including subscales; | **p:** Intervention group shows increases in coping attempts CSQ. Negative Thinking did not show sig. main effects. Analyses of the other CSQ scales suggested group differences over time for Pain Controllability, but not for Passive Adherence.; |
| | **s:** multilevel modeling of next day pain and same-day activity | **s:** simple slopes suggested that the use of smartphone-assisted skills on days with pain attenuated next-day pain versus no use of skills. Same-day intensity predicted sig. higher next-pain intensity. Interaction of skill use and activity engagement was not sig. |
| Irvine et al, 2015 [23] | **p:** back pain measures (pain, frequency, intensity, duration); Multidimensional Pain Inventory Interference Scale and Interference Scale of the Brief Pain Inventory (functionality and quality of life); Dartmouth CO-OP (function; well-being; and quality of life); worker productivity; presenteeism; catastrophizing of pain; | **p:** overall medium effect in treatment group versus control group: average back pain decreased sig. to baseline in treatment and sig. versus control group; overall sig. improvement in functionality, quality of life, and well-being in treatment versus control; regarding the worker productivity and the presenteeism measures, the overall tests were sig.; the overall tests for the catastrophizing of pain scale were n.s.; |
| | **a:** user satisfaction; website usability; understanding and implementation survey | **a:** higher satisfaction in treatment group; website usability was associated with *good to excellent* ratings; depending on the content medium to very high rates of understanding |
| Guillory et al, 2015 [22] | **p:** pain interference (activities, relations and sleep); affect rating (with photos); perception of social support; | **p:** sig. differences in pain interference between groups in general pain and interference with relations but not with sleep pain (was sig. at week 2-3); n.s. effect in affect rating (was sig. at week 2-3); no treatment effects for either social support or connectedness; sig. improvement in pain outcomes if stratified for married patients or patients in a partnership in intervention group versus control; |
| | **a:** feasibility | **a:** participants completed 79% of the measurements requested through the twice-daily reminder SMS[l] messages. 68% of participants completed ≥75% of the total measurements requested. The number of measurements completed did not differ between the intervention and control conditions. |

*If it was not mentioned differently in the study, outcomes were marked as primary outcomes. The exception was applicability outcomes. Applicability includes feasibility and satisfaction.

[a]a: applicability, p: primary, s: secondary

[b]sig: Significant(ly)

[c]PAM: Patient activation measure

[d]n.s.: No(t/n) significant(ly)

[e]VAMS: Visual analog moods scale

[f]NDI: Neck Disability Index

[g]FABQ: Fear-Avoidance Beliefs Questionnaire

[h]ROM: Range of motion

[i]PDI: Pain Disability Index

[j]HADS: Hospital Anxiety and Depression Scale

[k]CSQ: Coping Strategies Questionnaire for Sickle Cell Disease

[l]SMS: Short message service

**References:**


21. Blödt S, Pach D, Eisenhart-Rothe SV, Lotz F, Roll S, Icke K, Witt CM. Effectiveness of app-based self-acupressure for women with menstrual pain compared to usual care: a randomized pragmatic trial. Am J Obstet Gynecol 2018 Feb; 218(2):227.e1-227.e9
22. Guillory J, Chang P, Henderson CR, Shengelia R, Lama S, Warmington M, Jowza M, Waldman S, Gay G, Reid MC. Piloting a Text Message-based Social Support Intervention for Patients With Chronic Pain: Establishing Feasibility and Preliminary Efficacy. Clin J Pain 2015 Jun; 31(6):548-56
23. rvine AB, Russell H, Manocchia M, Mino DE, Cox GT, Morgan R, Gau JM, Birney AJ, Ary DV. Mobile-Web app to self-manage low back pain: randomized controlled trial. J Med Internet Res 2015 Jan 02; 17(1):e1
24. Schatz J, Schlenz AM, McClellan CB, Puffer ES, Hardy S, Pfeiffer M, Roberts CW. Changes in coping, pain, and activity after cognitive-behavioral training: a randomized clinical trial for pediatric sickle cell disease using smartphones. Clin J Pain 2015 Jun; 31(6):536-47
25. Skrepnik N, Spitzer A, Altman R, Hoekstra J, Stewart J, Toselli R. Assessing the Impact of a Novel Smartphone Application Compared With Standard Follow-Up on Mobility of Patients With Knee Osteoarthritis Following Treatment With Hylan G-F 20: A Randomized Controlled Trial. JMIR Mhealth Uhealth 2017 May 09; 5(5):e64
26. Stinley N, Norris D, Hinds P. Creating Mandalas for the Management of Acute Pain Symptoms in Pediatric Patients. Art Therapy 2015 Jun 22; 32(2):46-53
27. Sun Y, Jiang F, Gu JJ, Wang YK, Hua H, Li J, Cheng Z, Liao Z, Huang Q, Hu W, Ding G. Development and Testing of an Intelligent Pain Management System (IPMS) on Mobile Phones Through a Randomized Trial Among Chinese Cancer Patients: A New Approach in Cancer Pain Management. JMIR Mhealth Uhealth 2017 Jul 25; 5(7):e108
28. Guétin S, Brun L, Deniaud M, Clerc J, Thayer JF, Koenig J. Smartphone-based Music Listening to Reduce Pain and Anxiety Before Coronarography: A Focus on Sex Differences. Altern Ther Health Med 2016 Jul; 22(4):60-3
29. Guétin S, Diego E, Mohy F, Adolphe C, Hoareau G, Touchon J, Thayer Jf, Koenig J. A patient-controlled, smartphone-based music intervention to reduce pain—A multi-center observational study of patients with chronic pain. European Journal of Integrative Medicine 2016 Jun; 8(3):182-187
30. Jamison RN, Mei A, Ross EL. Longitudinal trial of a smartphone pain application for chronic pain patients: Predictors of compliance and satisfaction. J Telemed Telecare 2018 Feb; 24(2):93-100
31. Jibb LA, Stevens BJ, Nathan PC, Seto E, Cafazzo JA, Johnston DL, Hum V, Stinson JN. Implementation and preliminary effectiveness of a real-time pain management smartphone app for adolescents with cancer: A multicenter pilot clinical study. Pediatr Blood Cancer 2017 Oct; 64(10)
32. Lee M, Lee SH, Kim T, Yoo H, Kim SH, Suh D, Son J, Yoon B. Feasibility of a Smartphone-Based Exercise Program for Office Workers With Neck Pain: An Individualized Approach Using a Self-Classification Algorithm. Arch Phys Med Rehabil 2017 Dec; 98(1):80-87
33. Oldenmenger WH, Baan MAG, van der Rijt CCD. Development and feasibility of a web application to monitor patients' cancer-related pain. Support Care Cancer 2018 Feb; 26(2):635-642
34. Raj SX, Brunelli C, Klepstad P, Kaasa S. COMBAT study - Computer based assessment and treatment - A clinical trial evaluating impact of a computerized clinical decision support tool on pain in cancer patients. Scand J Pain 2017 Oct; 17:99-106
35. Huber S, Priebe JA, Baumann K, Plidschun A, Schiessl C, Tölle TR. Treatment of Low Back Pain with a Digital Multidisciplinary Pain Treatment App: Short-Term Results. JMIR Rehabil Assist Technol 2017 Dec 04; 4(2):e11
