# Supplementary material for: Benefits of Mobile Apps in Pain Management: Systematic Review
Source: JMIR Mhealth Uhealth. 2018 Oct 22;6(10):e11231. doi: 10.2196/11231 (PMC6231845; doi:10.2196/11231)
Supplement: Multimedia Appendix 5 [file mhealth_v6i10e11231_app5.pdf]

Multimedia Appendix 5: SIGN checklist for randomized controlled trials.

**Author, year**	**1.1**	**1.2**	**1.3**	**1.4**	**1.5**	**1.6**	**1.7**	**1.8 I[a]/C[b]/A[c] (%)**	**1.9**	**1.10**	**2.1**	**2.2**	**2.3**
Blödt et al, 2018 [21]	Y[d]	Y	Y	D[e]	N[f]	Y	Y	14.4/4.5	Y	D	+	CS[g]	Y
Sun et al, 2017 [27]	Y	Y	N	D	Y	Y	Y	0.0/0.0	Y	D	+	Y	Y
Skrepnik et al, 2017 [25]	Y	Y	Y	D	Y	Y	Y	2.8/0.96	CS	CS	+	Y	Y
Stinley et al, 2015 [26]	Y	Y	N	D	CS	Y	Y	D	Y	D	+	Y	Y
Schatz et al, 2015 [24]	Y	Y	Y	D	Y	Y	Y	0.0/8.0	Y	Y	++	Y	Y
Irvine et al, 2015 [23]	Y	CS	N	D	Y	Y	Y	4.5/1.5/1.5	Y	D	+	Y	Y
Guillory et al, 2015 [22]	Y	CS	N	D	Y	Y	Y	14.6/19.5	N	CS	+	Y	Y

[a]I: Intervention

[b]C: Control

[c]A: Alternative

[d]Y: Yes

[e]D: Does not apply

[f]N: No

[g]CS: Can't say

References:

21. Blödt S, Pach D, Eisenhart-Rothe SV, Lotz F, Roll S, Icke K, Witt CM. Effectiveness of app-based self-acupressure for women with menstrual pain compared to usual care: a randomized pragmatic trial. Am J Obstet Gynecol 2018 Feb; 218(2):227.e1-227.e9

22. Guillory J, Chang P, Henderson CR, Shengelia R, Lama S, Warmington M, Jowza M, Waldman S, Gay G, Reid MC. Piloting a Text Message-based Social Support Intervention for Patients With Chronic Pain: Establishing Feasibility and Preliminary Efficacy. Clin J Pain 2015 Jun; 31(6):548-56

23. rvine AB, Russell H, Manocchia M, Mino DE, Cox GT, Morgan R, Gau JM, Birney AJ, Ary DV. Mobile-Web app to self-manage low back pain: randomized controlled trial. J Med Internet Res 2015 Jan 02; 17(1):e1

24. Schatz J, Schlenz AM, McClellan CB, Puffer ES, Hardy S, Pfeiffer M, Roberts CW. Changes in coping, pain, and activity after cognitive-behavioral training: a randomized clinical trial for pediatric sickle cell disease using smartphones. Clin J Pain 2015 Jun; 31(6):536-47

25. Skrepnik N, Spitzer A, Altman R, Hoekstra J, Stewart J, Toselli R. Assessing the Impact of a Novel Smartphone Application Compared With Standard Follow-Up on Mobility of Patients With Knee Osteoarthritis Following Treatment With Hylan G-F 20: A Randomized Controlled Trial. JMIR Mhealth Uhealth 2017 May 09; 5(5):e64

26. Stinley N, Norris D, Hinds P. Creating Mandalas for the Management of Acute Pain Symptoms in Pediatric Patients. Art Therapy 2015 Jun 22; 32(2):46-53

27. Sun Y, Jiang F, Gu JJ, Wang YK, Hua H, Li J, Cheng Z, Liao Z, Huang Q, Hu W, Ding G. Development and Testing of an Intelligent Pain Management System (IPMS) on Mobile Phones Through a Randomized Trial Among Chinese Cancer Patients: A New Approach in Cancer Pain Management. JMIR Mhealth Uhealth 2017 Jul 25; 5(7):e108
